# Characterization of β-cell function and insulin resistance in overweight Chinese adolescents with normal glucose tolerance

**DOI:** 10.3892/etm.2013.1164

**Published:** 2013-06-17

**Authors:** MINGXIAO SUN, XIUQING HUANG, LEI JIANG, YI YAN, BOWEN LI, WEIJUAN ZHONG, JUNFEI CHEN, YIMIN ZHANG, ZHENGZHEN WANG, JIAN LI, MINHAO XIE

**Affiliations:** 1 Department of Endocrinology, Beijing Hospital, Beijing 100730;; 2 Beijing Institute of Geriatrics, Beijing Hospital, Beijing 100730;; 3 Sport Science College, Beijing Sport University, Beijing 100084, P.R. China

**Keywords:** insulin resistance, islet cell, secretion, adolescent, overweight

## Abstract

This study aimed to investigate the characteristics of insulin resistance and β-cell secretion in healthy adolescents. A total of 124 adolescents with normal glucose tolerance (NGT) were divided according to BMI into normal weight (n=31; control), overweight (n=52) and obese (n=41) groups. Oral glucose tolerance tests were performed, and blood glucose (G_0_, G_30_ and G_120_) and insulin (I_0_, I_30_ and I_120_) levels at 0, 30 and 120 min, respectively, were measured. The homeostasis model assessment estimate of insulin resistance (HOMA-IR) and early insulin release index (IRI) were calculated to evaluate insulin sensitivity and early β-cell secretion. The G_0_, G_120_ levels and the natural logarithm (Ln) of I_30_ and ΔI_30_/ΔG_30_ were similar in the overweight and obese groups, but significantly higher compared with those of the normal weight group (P<0.05). LnI_0_ and LnHOMA-IR progressively increased (P<0.01) in correlation with the degree of obesity among the three groups. LnΔI_30_/ΔG_30_ and LnHOMA-IR were significantly positively correlated with the indices of obesity (P<0.001 and P<0.05, respectively). LnHOMA-IR was also positively correlated with the insulin levels at 30 and 120 min (r=0.454 and 0.314, respectively; P<0.001). In healthy adolescents, insulin resistance progressively increased with increased body mass index (BMI), but the compensatory increase in early insulin secretion was limited.

## Introduction

Since the 1990s, the proportions of overweight and obese Chinese adolescents have significantly increased (1). The prevalence of obesity in adolescents contributes to the early onset of obesity-associated diseases, including type 2 diabetes, hypertension and dislipidemia. The prevalence of obesity continues to increase (2,3). Obesity is the main risk factor for the early onset of type 2 diabetes. Insulin resistance (IR) and defects in islet β-cell insulin secretion are the main causes of impaired glucose tolerance (IGT) and overt type 2 diabetes in obese adults and adolescents (4–6).

In adults with normal glucose tolerance (NGT), the various extents of IR and insulin secretion have previously been identified between normal weight and obese individuals (7,8). Numerous studies have focused on metabolic abnormalities in adolescents with diabetes and IGT (9,10). However, among Asian adolescents with NGT, the changes in IR and insulin secretion remain unclear. We hypothesize that differences exist in the IR and secretion among normal weight, overweight and obese adolescents with NGT.

## Subjects and methods

### Subjects

A total of 124 adolescents (59 males and 65 females, mean age plus or minus standard deviation=13.6±0.7 years) from a high school (27th High School, Beijing, China) volunteered to participate in this study. The volunteers were asked to answer a questionnaire concerning their health and family history under the direction of the doctors. Maturational status was divided into pubertal and prepubertal based on the onset of menarche in girls and semen production in boys. After explaining the study procedures and protocol to the participants and their parents, informed consents were obtained prior to the initiation of the study. The study was approved by the Ethics Committee of the Beijing Sport University (Beijing, China) and the Ministry of Education (Beijing, China).

The subjects were divided into three groups according to their body mass index (BMI): the obese (n=41, BMI=24.7–34.1 kg/m^2^), overweight (n=52, BMI=22.4–27.7 kg/m^2^) and normal weight (n=31, BMI=14.6–21.9 kg/m^2^) groups. The age- and gender-specific BMI cutoff points used in this study were those defined by the Group of China Obesity Task Force (11). The subjects were not taking any medications and had not previously suffered from any disease known to influence body composition, insulin sensitivity, physical activity or dietary intake.

### Anthropometry and measurement of body composition

The measurements included anthropometry (height, body weight and BMI) and fat distribution (percentage body fat and percentage truncal fat). Body weight and height were measured to an accuracy of 0.1 kg and 0.1 cm, respectively. BMI was calculated as the body weight in kilograms divided by the square of the height in centimeters. Body and truncal fat were measured by dual-energy X-ray absorptiometry (Lunar DPX-L; Lunar Corporation, Madison, WI, USA) and expressed as a percentage.

### Oral glucose tolerance test (OGTT), IR and secretion

Each participant was subjected to a 120 min OGTT (dextrose, 1.75 g/kg body weight; up to 75 g) after 10 h of overnight fasting (12). Venous blood samples were obtained at 0, 30 and 120 min to determine the plasma glucose levels (G_0_, G_30_ and G_120_) using the glucose oxidase method (Beckman, Albertville, MN, USA), and the insulin levels (I_0_, I_30_ and I_120_) using chemiluminescence (DPC Immulite, Los Angeles, CA, USA), respectively.

The homeostasis model assessment estimate of IR (HOMA-IR) was calculated from fasting insulin (I_0_) and fasting glucose (G_0_) as follows: HOMA-IR=(I_0_×G_0_)/22.5, with insulin levels in *μ*U/ml and glucose levels in mmol/l (13). First-phase insulin secretion was estimated using the following index equation: early insulin release index (IRI)=Δ30 insulin (I_30_−I_0_)/Δ30 glucose (G_30_−G_0_). The IRI is an indicator of β-cell function (9).

### Statistical analyses

All variables were examined for normalities of distribution by the Kolmogorov-Smirnov test. Due to the abnormal distributions, the insulin levels and associated data were subjected to natural logarithm transformation (expressed by LnI_0_, LnI_30_, LnI_120_, LnHOMA-IR and LnIRI). Continuous measurement data are expressed as the mean ± standard deviation. Comparisons among groups were evaluated using one-way ANOVA and the differences were further evaluated using Fisher’s least significant difference test. Numerical data were expressed as rates and compared using the χ^2^ test. Spearman’s correlation coefficients were used to determine bivariate relation for either two parameters. P<0.05 was considered to indicate a statistically significant result. All data were analyzed with SPSS 17.0 software (SPSS, Inc., Chicago, IL, USA).

## Results

The girls in this study were more mature than the boys (prepubertal/pubertal; 3/58 in girls vs. 30/21 in boys, P<0.0001). Twelve adolescents (4 girls and 8 boys) failed to report their maturational status. The girls had a significantly higher percentage of body and truncal fat compared with boys in all three groups (data not shown). No significant gender difference was observed in LnHOMA-IR and LnIRI. Therefore, data from the girls and boys were combined for all other analyses.

Table I shows significant differences in anthropometric measurements and the percentage of fat among the normal weight, overweight and obese groups. By contrast, no significant difference was observed in age, gender, birth weight, maturational status and family history of type 2 diabetes among the three groups.

**Figure 1.**
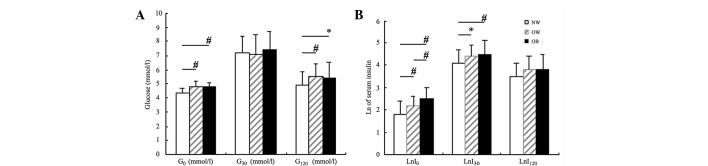
Comparisons of blood glucose and natural logarithm (Ln) of insulin levels among the three groups. (A) Comparison of blood glucose levels at fasting, 30 and 120 min (G_0_, G_30_ and G_120_, respectively) via separate oral glucose tolerance tests among the three groups: NW, normal weight group; OW, overweight group; OB, obese group. (B) Comparison of Ln of blood insulin levels at fasting, 30 and 120 min (LnI_0_, LnI_30_ and LnI_120_, respectively) via separate oral glucose tolerance tests among the three groups: NW, normal weight group; OW, overweight group; OB, obese group. ^*^P<0.05, ^#^P<0.01.

Fig. 1A shows that the blood glucose levels of all subjects at various time points were within the normal range. The blood glucose levels at various time points were not significantly different between the overweight and obese groups. However, the blood glucose levels at 0 and 120 min of these two groups were both higher than those of the normal weight group (P<0.001 and P<0.05), whereas no significant difference was observed in the 30 min glucose levels among the three groups (P>0.05).

Fig. 1B shows that with increased severity of obesity, LnI_0_ gradually increased among the three groups (P<0.01). LnI_30_ was not significantly different between the overweight and the obese groups, but was higher than that of the normal weight group (P<0.05 and P<0.001). LnI_120_ was similar among all three groups.

**Figure 2.**
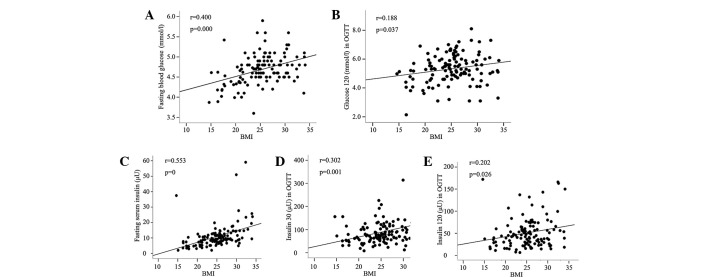
Correlation analyses between variables in OGTT and BMI. Correlation analyses between BMI and (A) fasting glucose, (B) glucose at 120 min in the OGTT, (C) fasting serum insulin level and insulin levels at (D) 30 min and (E) 120 min in the OGTT. r, correlation coefficient; OGTT, oral glucose tolerance test; BMI, body mass index.

Fig. 2 shows that the BMI was positively correlated with the G_0_ and G_120_ levels. The BMI was also positively correlated with the insulin levels at three time points (P<0.05). The percentage of body and truncal fat was observed to have a similar correlation with G_0_, G_120_ and insulin levels (r=0.268–0.452, P<0.001–0.003). With regard to the association between blood glucose and insulin levels, each corresponding time point (0 to 0, 30 to 30, and 120 to 120 min) exhibited a significant positive correlation (P<0.01), and the blood glucose level at 30 min was also correlated with the insulin level at 120 min (r=0.282, P=0.002).

**Figure 3.**
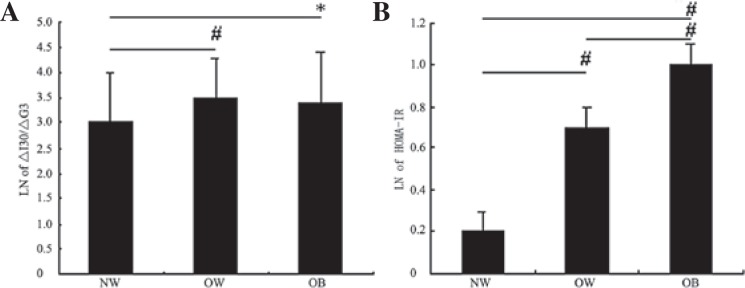
Comparisons of IRI and HOMA-IR among the three groups. (A) Comparison of natural logarithm (Ln) of ΔI_30_/ΔG_30_ among the three groups: NW, normal weight group; OW, overweight group; OB, obese group. (B) Comparison of Ln of HOMA-IR among the three groups: NW, normal weight group; OW, overweight group; OB, obese group. ^*^P<0.05, ^#^P<0.01. IRI, insulin release index; HOMA-IR, homeostasis model assessment estimate of insulin resistance; ΔI_30_/ΔG_30_=(I_30_−I_0_)/(G_30_−G_0_); I_0_, fasting insulin level; I_30_, insulin level at 30 min; G_0_, fasting glucose level; G_30_, glucose level at 30 min.

LnIRI in the normal weight group was significantly lower than that in the overweight and obese groups (P<0.05), whereas no significant difference was observed between the latter two groups (Fig. 3A). Fig. 3B shows that among the three groups, the degree of IR increased with increased severity of obesity (P<0.01).

**Figure 4.**
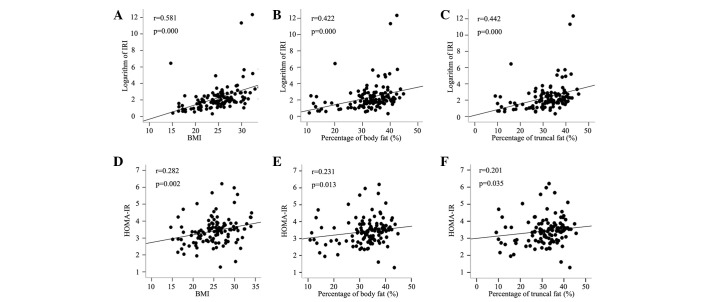
Correlation analyses between variables and HOMA-IR, as well ΔI_30_/ΔG_30_ (IRI). Correlation analyses between LnIRI and (A) BMI, (B) percentage of body fat and (C) percentage of truncal fat. Correlation analyses between HOMA-IR and (D) BMI, (E) percentage of body fat and (F) percentage of truncal fat. r, correlation coefficient; HOMA-IR, I_0_ (*μ*U/ml) × G_0_ (mmol/l)/22.5; ΔI_30_/ΔG_30_=(I_30_−I_0_)/(G_30_−G_0_); I_0_, fasting insulin level; I_30_, insulin level at 30 min; G_0_, fasting glucose level; G_30_, glucose level at 30 min; IRI, insulin release index; Ln, natural logarithm; BMI, body mass index.

Fig. 4 shows that HOMA-IR and IRI were positively correlated with BMI as well as truncal and body fat percentages. HOMA-IR was significantly correlated with the 30 and 120 min insulin levels (r=0.454 and 0.314, respectively; P<0.001), except for the fasting blood glucose and insulin level. ΔI_30_/ΔG_30_ was not correlated with the blood glucose or insulin levels at 120 min, with the exception of fasting blood glucose, fasting and 30 min insulin levels.

## Discussion

Insulin is an important hormone that maintains blood glucose in homeostasis. The secretion of insulin is regulated by the blood glucose level. With decreased insulin sensitivity caused by IR, the islet β cells maintain the blood glucose in a stable state through a compensatory increase in insulin secretion. With increased dedifferentiation of β cells, early decompensation involving increased glucose levels occurs (13). The main cause of the early onset of type 2 diabetes in adolescents has been shown to be the marked loss of β-cell function (6). However, numerous studies on adolescents have demonstrated that in the overweight or obese stage, the β-cell function remains stable or exhibits higher activity even with impaired glucose metabolism (14,15). β-cell hyperactivity, with increased pancreatic β-cell mass that may be associated with increased free fatty acids, contributes to insulin oversecretion in response to IR in obese individuals (15).

To the best of our knowledge, the current study is the first to reveal the characteristics of IR and β-cell function on glucose regulation in normal weight, overweight and obese Asian adolescents. Our data suggests that IR exists in over-weight and obese adolescents, and the degree of IR correlates with the severity of obesity. Islet β cells are able to maintain a normal blood glucose level in overweight adolescents through increased early insulin secretion. However, once the body weight reaches the obesity stage, further insulin secretion does not occur.

We observed that the glucose levels at fasting and 120 min after glucose challenge were higher in overweight and obese adolescents, and were positively correlated with BMI as well as body and truncal fat percentages, although glucose tolerance was normal in all subjects. These results indicated that the increase in blood glucose levels, which was associated with the increase in body weight and adipose tissue, emerged in the euglycemic stage, even though the weight gain was only maintained in the overweight stage.

To further understand the increase in glucose at the early stages of becoming overweight, we analyzed the levels of IR and early insulin secretion. IR is a main feature of obesity that reportedly exists persistently throughout the entire disease process of type 2 diabetes (10). HOMA-IR has been demonstrated to be a feasible method of estimating insulin sensitivity in prepubertal and pubertal obese subjects (16). As expected, insulin sensitivity was clearly observed in the overweight and obese groups. We demonstrated that the fasting insulin levels and HOMA-IR progressively increased with increased obesity. IR has been demonstrated to be the main prognostic factor for IGT in obese adolescents (17). The prevalence of impaired glucose metabolism escalates in overweight adolescents, even at minimally overweight levels, and is associated with pronounced deterioration of insulin sensitivity (14). Correlation analysis revealed that fasting glucose was positively correlated with HOMA-IR but not with IRI, and the coefficient correlation of fasting glucose with BMI was also higher than that of the other time point of glucose levels with BMI. We suggest that the higher fasting glucose levels in overweight and obese groups occurred in the early normal glycometabolism stage, and were mainly caused by the reduced insulin sensitivity associated with increased obesity. This result was in accordance with the study by O’Malley *et al* (17), which suggests that in obese youth, insulin sensitivity declines when the normal fasting plasma glucose level changes from low to high. In addition, the odds of presenting with IGT were reported to increase by 4.5% with each 0.06 mmol/l increase in fasting plasma glucose.

Our data showed that although higher fasting glucose levels existed in the overweight and obese groups, the 30 min glucose levels demonstrated no difference among the three groups. We observed that the IR-associated 30 min insulin secretion increased in the overweight and obese groups. This observation may be attributed to a compensatory mechanism under the reduced insulin sensitivity, which maintained the higher level of early IRI, thereby ensuring that the 30 min blood glucose levels were consistent with those of the normal-weight group.

IR progressively increased with increased obesity. However, the IRI did not further increase in the obese group, and the 120 min insulin levels in all three groups returned to similar levels. These results may explain the higher blood glucose levels at 120 min in the overweight and obese groups compared with those of the normal group. These findings also suggest that when the compensatory capacity reached a certain degree, islet β-cell secretion did not further increase to offset the effect of insulin sensitivity deterioration and to maintain blood glucose in a stable state.

Our results were consistent with those of a study on obese youth with NGT by Yeckel *et al* (18). Yeckel *et al* showed that increased 120 min plasma glucose within the NGT range was associated with a specific impairment in β-cell responsiveness distinct from the deterioration of insulin sensitivity.

Notably, the 30 min glucose and 120 min insulin levels in the overweight and obese groups did not differ from those in the normal weight group. However, a positive correlation existed between these two variables. Therefore, with the exception of the early-phase compensation insulin release, delayed second-phase secretion may also exist in healthy overweight adolescents in addition to the impaired glucose regulation in youth (19).

In summary, the fasting and 120 min blood glucose levels following glucose challenge were higher in overweight and obese adolescents than in normal weight adolescents, although the glucose levels were all within the normal range. IR played an important role in glucose regulation throughout the process from the overweight to the obese stage and may be a hallmark feature of obesity in adolescents.

Although islet β-cell secretion increased in the overweight stage, the degree of compensatory increase in insulin secretion reached a plateau, even with increased obesity. This phenomenon may explain the development of impaired glucose regulation in obese adolescents. Therefore, the prevention of type 2 diabetes in adolescents should start in the early over-weight stage when the glucose metabolism is normal.

## Figures and Tables

**Table I.** Subject characteristics.

Variable	Group			P-value		
	NW	OW	OB	P1	P2	P3
Number of cases	31	52	41			
Age (years)^a^	13.6±0.6	13.7±0.6	13.1±0.8	0.860	0.417	0.265
Male/female	13/18	22/30	24/17	0.672	0.264	0.084
Prepubertal/pubertal	8/20	13/35	12/24	0.889	0.683	0.535
Birth weight (kg)^a^	3.4±0.6	3.4±0.5	3.4±0.6	0.991	0.868	0.838
Family history of DM (Y/N)	13/18	17/35	15/26	0.396	0.645	0.695
BMI (kg/m^2^)^a^	19.3±2.2	24.9±1.3	29.7±2.4	<0.001	<0.001	0.000
Body fat (%)^a^	24.5±8.7	33.8±4.9	36.6±7.5	<0.001	<0.001	0.022
Truncal fat (%)^a^	21.9±8.8	33.3±5.6	36.2±4.3	<0.001	<0.001	0.033
Waist circumference (cm)^a^	65.2±4.8	81.8±7.1	94.6±7.1	<0.001	<0.001	<0.001
Hip circumference (cm)^a^	85.8±4.6	97.5±4.7	105.7±5.3	<0.001	<0.001	<0.001

NW, normal weight group; OW, overweight group; OB, obese group. P1, comparison between the overweight and normal weight groups; P2, comparison between the obese and normal weight groups; P3, comparison between the overweight and obese groups. DM, diabetes mellitus; Y, yes; N, no; BMI, body mass index.

a Mean ± SD.
